# Diagnostic accuracy of detection and quantification of HBV-DNA and HCV-RNA using dried blood spot (DBS) samples – a systematic review and meta-analysis

**DOI:** 10.1186/s12879-017-2776-z

**Published:** 2017-11-01

**Authors:** Berit Lange, Teri Roberts, Jennifer Cohn, Jamie Greenman, Johannes Camp, Azumi Ishizaki, Luke Messac, Edouard Tuaillon, Philippe van de Perre, Christine Pichler, Claudia M. Denkinger, Philippa Easterbrook

**Affiliations:** 1 Division of Infectious Diseases, Department of Medicine II, Medical Center – University of Freiburg, Faculty of Medicine, University of Freiburg, Freiburg, Germany; 2 Centre for Chronic Immunodeficiency, Medical Center – University of Freiburg, Faculty of Medicine, University of Freiburg, Freiburg, Germany; 3 FIND, Geneva, Switzerland; 4 Department of Infectious Diseases, University of Pennsylvania, Philadelphia, PA USA; 5 Global Hepatitis Programme, HIV Department, World Health Organization, Geneva, Switzerland; 6 Pathogenesis and Control of Chronic Infections UMR 1058, INSERM/Université Montpellier/Etablissement Français du Sang, INSERM, 34394, Cedex 5 Montpellier, France; 7 Centre Hospitalier Universitaire (CHU) de Montpellier, département de bactériologie-virologie, Montpellier, France

**Keywords:** Dried blood spots (DBS), HCV RNA, HBV DNA, Virological testing, Diagnostic accuracy

## Abstract

**Background:**

The detection and quantification of hepatitis B (HBV) DNA and hepatitis C (HCV) RNA in whole blood collected on dried blood spots (DBS) may facilitate access to diagnosis and treatment of HBV and HCV infection in resource-poor settings. We evaluated the diagnostic performance of DBS compared to venous blood samples for detection and quantification of HBV-DNA and HCV-RNA in two systematic reviews and meta-analyses on the diagnostic accuracy of HBV DNA and HCV RNA from DBS compared to venous blood samples.

**Methods:**

We searched MEDLINE, Embase, Global Health, Web of Science, LILAC and Cochrane library for studies that assessed diagnostic accuracy with DBS. Heterogeneity was assessed and where appropriate pooled estimates of sensitivity and specificity were generated using bivariate analyses with maximum likelihood estimates and 95% confidence intervals. We also conducted a narrative review on the impact of varying storage conditions or different cut-offs for detection from studies that undertook this in a subset of samples. The QUADAS-2 tool was used to assess risk of bias.

**Results:**

In the quantitative synthesis for diagnostic accuracy of HBV-DNA using DBS, 521 citations were identified, and 12 studies met the inclusion criteria. Overall quality of studies was rated as low. The pooled estimate of sensitivity and specificity for HBV-DNA was 95% (95% CI: 83–99) and 99% (95% CI: 53–100), respectively. In the two studies that reported on cut-offs and limit of detection (LoD) – one reported a sensitivity of 98% for a cut-off of ≥2000 IU/ml and another reported a LoD of 914 IU/ml using a commercial assay. Varying storage conditions for individual samples did not result in a significant variation of results. In the synthesis for diagnostic accuracy of HCV-RNA using DBS, 15 studies met the inclusion criteria, and this included six additional studies to a previously published review. The pooled sensitivity and specificity was 98% (95% CI:95–99) and 98% (95% CI:95–99.0), respectively. Varying storage conditions resulted in a decrease in accuracy for quantification but not for reported positivity.

**Conclusions:**

These findings show a high level of diagnostic performance for the use of DBS for HBV-DNA and HCV-RNA detection. However, this was based on a limited number and quality of studies. There is a need for development of standardized protocols by manufacturers on the use of DBS with their assays, as well as for larger studies on use of DBS conducted in different settings and with varying storage conditions.

## Background

Chronic hepatitis B (HBV) and C (HCV) virus infection are major causes of morbidity and mortality. The burden is particularly high in low and middle income settings, such as Sub-Saharan African, East Asia, Central Asia and the Middle East [1–3]. However, only a minority of those infected with HBV and HCV had been tested and knew their status: 9% of persons living with HBV (22 million) and 20% of persons living with HCV (14 million) [3]. This is largely due to limited availability and access to testing facilities and affordable diagnostic assays. Overall, it is estimated that between 10% to 40% of those who are hepatitis B surface antigen positive are in need of treatment [3]. While treatment is recommended in all persons with evidence of cirrhosis (based on either clinical assessment or use of non-invasive tests for staging of liver disease), in those without cirrhosis, assessment for treatment eligibility and treatment response requires access to quantitative measurement of HBV-DNA. In those without cirrhosis, treatment is recommended in all international guidelines for those persons with an HBV-DNA level of >2000 to 20,000 IU/ml and abnormal liver function tests [4–9]. However, access to HBV-DNA testing in low-income countries currently remains very limited.

Spontaneous clearance of acute HCV infection generally occurs within six months of infection in 15–45% of infected individuals in the absence of treatment. Therefore, once exposure to HCV is established with a positive anti-HCV antibody results, testing for viraemic HCV infection through detection of HCV RNA (using either quantitative or qualitative nucleic acid tests (NAT)) or HCV (p22) core antigen (HCVcAg) is performed to confirm chronic HCV infection and need for treatment. While all persons who are HCV RNA positive are eligible for treatment, in many settings criteria are applied so that those with more advanced disease are prioritized for treatment. It is also used to confirm cure based on HCV-RNA measurement 12 weeks after the end of treatment (defined as a sustained viral response) [5].

Significant scale up in access to hepatitis testing and treatment will require both further simplification of the process of diagnosis, and methods to facilitate access to testing, especially in remote settings, and among vulnerable populations, such as PWID and people in prison. DBS sampling is an alternative specimen collection method usually by capillary finger-stick (or heel-prick in infants), that does not require venepuncture, embedding the drops of blood onto filter paper, that can then be transported by posting to a laboratory, where testing can take place. DBS sampling is being increasingly used to expand access to diagnosis of viral infections in remote and under-resourced regions with limited access to laboratory services [10–13] and in particular for early infant diagnosis of HIV [14] and HIV viral load monitoring [15]. The simplicity and relative ease of specimen collection, preparation, transport and storage make DBS specimens a potential option for both serological testing and NAT in low-resource settings [10]. It may also reduce costs associated with sample collection, storage and transportation, and allow for batch testing in a centralized laboratory. The main disadvantage is that manufacturers have not developed standardised protocols for the use of DBS with their assays and therefore current use of DBS is off-label, and not approved by major regulatory authorities.

The first steps to measure HBV-DNA using DBS were made more than 20 years ago [16], but previous reviews evaluating DBS did not include HBV-DNA [15, 17, 18]. Our objective was to evaluate the diagnostic performance of DBS compared to venous blood samples for detection and quantification of HBV-DNA and impact of different storage conditions, and to update an existing systematic review on HCV-RNA [18]. This was undertaken alongside a companion systematic review evaluating the diagnostic performance of DBS for serological testing for HBV and HCV [19]. Both reviews were undertaken to inform recommendations on strategies to expand access to and uptake of hepatitis testing in the WHO 2017 hepatitis testing guidelines [20].

## Methods

### Search strategy and selection criteria

Following PRISMA reporting guidelines for systematic reviews 19) and QUADAS-2 criteria on assessment of risk of bias [21], we conducted a systematic review to evaluate use of DBS for testing and detection of HBV-DNA and HCV-RNA. We searched six databases (MEDLINE, Web of Science, EMBASE, Global Health, and Cochrane library and LILACS databases) on 1st September 2015 for English language studies that reported the diagnostic performance of DBS compared to venous blood samples for detection and quantification of HBV-DNA and HCV-RNA, published between 1970 and 2015. The search was updated on 22.8.17. The search terms used were (adapted to databases): DBS, Dried blood spot, filter paper, Guthrie paper, hepatitis, HBV, HCV, HBsAg, Hepatitis B, Hepatitis C, Hepatitis C RNA, Hepatitis B DNA.

Eligible studies were those that compared the index test HBV DNA/HCV RNA using DBS against a reference test for HBV-DNA or HCV RNA in serum/plasma and reported some or all of the following outcomes: correlation, regression coefficient, specificity, sensitivity and positive/negative predictive values. Index and reference assays used did not have to be the same. We included different types of study design, including cross-sectional and cohort studies. There were no date, geographic or population demographic restrictions, and individuals of all age groups were included.

### Screening and data extraction

Two independent reviewers screened all sources for title, abstract and full-text review to identify eligible studies using pre-defined eligibility criteria, with a third reviewer where there was uncertainty about inclusion or exclusion. The same data extraction procedure was performed in duplicate for every study and included the following variables: author, publication and study dates, country, percentage of children and adults, age range, gender distribution, type of specimen used for DBS, specimen used as gold standard (plasma or serum), test used, storage conditions and effect of storage conditions.

### Risk of bias and quality assessment

The categories in the QUADAS-2 tool relating to patient selection, performing index and reference test and flow and timing of the study were used and adapted to assess risk of bias of included studies [21]. We judged studies to be at low risk of bias, when consecutive sampling of patients was used, the conduct of the laboratory based test used standardized protocols for all samples and the patient flow and timing were well reported and clear for all patients/samples included. In contrast we considered studies to be at high risk of bias in these categories if either a case control design was used, or conduct of diagnostic tests was not clearly reported or if patient flow was not reported or inconsistent.

### Statistical data analysis

Summary estimates of sensitivity and specificity were generated using a bivariate random effects meta-analysis using maximum likelihood estimate and 95% confidence intervals when there were more than 4 studies contributing to the analysis. Positive (Sensitivity/(1-Specificity) and Negative (1-Sensitivity/Specificity) likelihood ratios were calculated directly from the pooled sensitivity and specificity. If studies did not have sufficient quantitative data (for example no samples with a negative reference test) to contribute to both sensitivity and specificity, we performed a univariate random effects meta-analysis of the sensitivity or specificity estimates separately.

We visually assessed forest plots for heterogeneity across the studies. We also report on estimates of τ2 and its *p*-value corresponding to the variance of the logit-transformed specificity and sensitivity as a measure of between-study variability [22]. Statistical analysis of the data was performed using STATA 14 (StataCorp. 2015. Stata Statistical Software: Release 14. College Station, TX: StataCorp LP). We assessed studies that exposed subsets of samples to varying storage conditions or were used to calculate or define cut-offs or limit of detection and these were included in a narrative analysis.

## Results

### HBV-DNA using DBS samples

#### Included studies

**Fig. 1:**
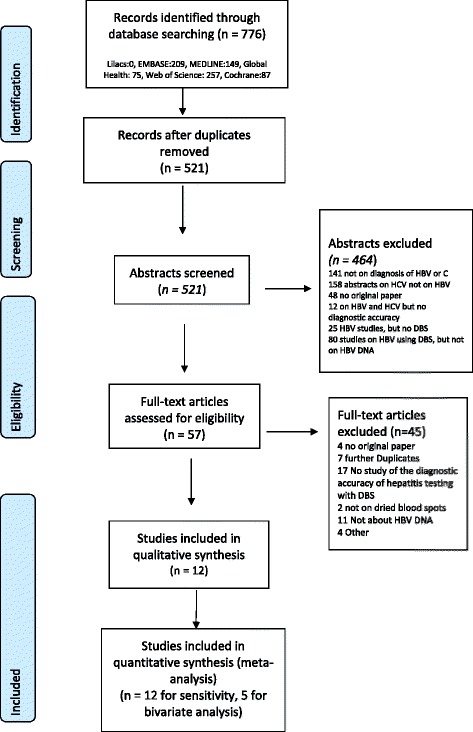
Prisma flow chart of studies for the systematic review of detection of hepatitis B DNA from DBS samples compared to venous blood sampling (plasma/serum)

After de-duplication we identified 521 abstracts for screening. 57 full-text papers were screened for potential inclusion and 12 studies were eligible for inclusion in the qualitative review [16, 23–33] (Fig. 1 and Table 1). Only five studies contributed to both sensitivity and specificity estimates (see Table 1). Five were from Europe (France [27], Denmark [30], Germany [32, 33], Spain [25]), four were from Africa (Ethiopia [31],Congo [23], Egypt [24] and Zambia [28]) two from India [16, 29], and one from Mexico [26]. All studies used whole blood apart from one that used plasma [23] for preparation of DBS or dried plasma spot (DPS) samples. One study included data on only women [16] and another only on HIV positive persons [28]. There were no data from children. Overall studies under-reported demographic characteristics.

**Table 1:** Characteristics of studies included in the systematic review of detection of HBV-DNA and update of the systematic review on HCV-RNA from DBS samples compared to venous blood sampling (plasma/serum)

Author, Country, Year	Journal	Study pop and sample size	Storage conditions	DBS collection method	Plasma method PCR	DBS method PCR	Limit of detection	Specificity	Sensitivity	Correlation/Association	Effect of storage conditions	Comments
Alidjinou Congo 2014	Diagnostic Microbiology and Infectious Disease	32 HBV patients	DBS at room temperature, Frozen plasma samples at −80 °C	30 μl of plasma onto filter paper	COBAS Taqman/COBAS AmpliPrep	COBAS Taqman/COBAS AmpliPrep	Detection limit for plasma was 12 IU/ml, In 3 patients, viral load in plasma was 152, 250, and 1727 IU/mL, respectively, whereas HBV-DNA could not be quantified in DPS, but was detected	NR	25/26 96%	Spearman correlation coefficient *r* = 0.84	NR	NR
Alhusseini Egypt 2012	Americal Journal of Biochemistry and Biotechnology	50 HBs Ag pos, but only 42 later included in accuracy calculation10 negative controls	Stored at −80 °C	50 μl from whole blood sample on Watman filter paper	In house	In house	No cut off suggested,	10/10 100%	42/42 100%	Good correlation (*r* = 0.88) between DBS and plasma viral load	NR	NR
Durgadevi India 2012	Conference abstract	60 hospital patients, 30 Hep B surface marker positive 22 of which were HBV-DNA pos by serum ELISA and 30 negative	DBS were stored at 25° for 4 and 7 days	NR	NR	NR	NR	NR	22/22100%	NR	All 22 serum positive cases could be detected from DBS after 4 and after 7 days at 25°	NR
Gupta India 1992	J Clin Microbiology	60 mothers with chronic HBV infection, 5 known negative laboratory staff	−20C for filter paper	5 μl whole blood from finger prick on Whatman paper	In house (endpoint)	In house (endpoint)	Limit of detection 10E4 virus particles in each 5 μl blood spot	13/15 86% 5/5 lab staff	43/45 96%	NR	Complete stability was maintained at 37 °C for 5 months, less denaturated protein when stored at 37 °C	No diagnostic accuracy calculation but can be calculated from data
Gruner 2015 Germany	Journal of Visualized Experiments	Inpatients, 299	Temperature: −20C, 4C or ambient temperature Time: Up to 14 days	Venipuncture, 100 μl whole blood on paper card	Not specified	Not specified	Unclear, but 7 samples not detected with DBS had HBV DNA concentrations between 409 to 3643 IU/ml	NR	93% 93/100	NR	NR	NR
Jardi Spain 2004	Hepatology	82 patients with chronic HBV infection (23 HBeAgpos, 39 HBeAgneg, 20 HBeAg inactive, 15 HBe neg under lamivudine therapy	Storage at −20 °C	20 μl of capillary blood on 5 mm paper disks (Scheicher)	In house	In house	LOD 100cop/ml among eight samples with serum HBV DNA between 10E3 and 10E4 copies/mL, seven tested positive using DBS samples, and among four samples with detectable serum HBV-DNA levels _10E3 copies/mL, none were positive using DBS samples.	NR	72/77 94%	Regression coefficient HBV-DNA concentration in DBS versus serum samples r(2) = 0.96 (*P* < .001).	Stability of unknown number of DBS samples assessed by leaving samples for several days in different conditions, no effect on HBV-DNA levels	NR
Lira Mexico 2009	Virology Journal	47 HBV HBsAg positive patients	Plasma samples at −70 °C, filter paper at −20 °C	50 μl per card (Schleicher + Schull)	QIAamp® Ultrasens® Virus kit (QIAGEN GMBH, Germany),	NR	NR	NR	47/47100%	The Pearson correlation 0.93 (*p* = 0.01)	No adverse effect by sample storage from ten patients at 4 degree, 25 degree, and 37 degree C for 7 days	NR
Mohamed France 2013	PLOS One	50 HBV positive patient, 10 HBV negative patients	Dried for 18 h in room temperature, then processed	50 μl on 12 mm discs (Whatman)	Cobas AmpliPrep/Cobas Taqman HBV test,	Cobas AmpliPrep/Cobas Taqman HBV test,	limit of detection of HBV-DNA 20 IU/ml plasma, limit of detection DBS 914 IU/ml	49/50 98%	50/50100%	Correlation good between DBS and plasma (r2 = 0.86, *p* < 0.001),	No difference in HBV-DNA levels at 1,3,7 and 14 days of storage at room temperature	Lower sensitivity in patients with 1000 IU/ml
Mossner Denmark 2016	Cohort	404 Prospective patients from hepatitis clinic and blood donors	Temperature: Room temperature Time 1–5 days	Finger prick, 75 μ on Whatman filter paper	HBV DNA (Ultrio Elite dHBV). COBAS® AmpliPrep/COBAS® TaqMan® 48 system (Roche Diagnostics, Basel, Switzerland)	HBV DNA (Ultrio Elite dHBV). COBAS® AmpliPrep/COBAS® TaqMan® 48 system (Roche Diagnostics, Basel, Switzerland)	Limit of detection for HBV-DNA 200 IU/ml	7/7 100%	53/76 70,7%	NR	Variation of 24 h to up to 7 d found no difference in stability of samples	
Ross Germany 2013	Virology	150 samples	Dried overnight at room temperature, then processed	100 μl applied to filter paper (Whatman, Schleicher + Schüll)	artus HBV LC PCR (Qiagen, Hilden, Germany)	artus HBV LC PCR (Qiagen, Hilden, Germany)	Limit of detection 100 iU/ml in plasma, 7 samples with HBV concentrations of 409–3643 IU/ml missed	50/50100%	93/100 93%	NR	NR	NR
Stene Johansen Ethiopia 2016	PLOS One	Known HBs pos patients, recruited at hospital	Dried overnight, then stored at ambient temperatures for a median of 24 days	Venipuncture, 80 μl applied to Whatman filter card	Abbott RealTime HBV assay).	Abbott RealTime HBV assay).	NR	NR	21/21 100% (cut off <2.7 log IU/ml) 23/26 88.5% (for all plasma detectable HBV DNA)	*R* = 0.92	NR	NR
Vinikoor Zambia 2015	Clinical Journal of Virology	68 HBs pos patients,	Dried for 12 hours at room temperature, stored at -80C	50 μl applied to Whatman filter paper	Cobas AmpliPrep/Cobas Taqman HBV test	Cobas AmpliPrep/Cobas Taqman HBV test	The probability of a undetectable DBS resultat a plasma viral load of 200 IU/ml was 13.8% (95% CI: 7.7–23.7) but this dropped to 1.8% (95% CI: 0.5–6.6) when a 2000 IU/ml cut-off was used and 0.2% (95% CI: 0.03–1.7) at 20,000 IU/ml.	62/68 91%	NR	NR	NR	NR
HCV-RNA – additional studies from upgrade of existing systematic review [18]												
Dokubo US 2014	Journal of Clinical Virology	148 participants in a prospective study of HCV	DBS air-dried for 2 hours, then sent to another insttute, then stored at −70 °C	Fingerstick on Whatman 903 cards 0.5 ml blood	Standard diagnostics HCV TMA (Norvatis®)	Standard diagnostics HCV TMA (Norvatis®)		100% 84/84	90% 43/48	Kappa = 0.92	NR	
Gruner 2015 Germany	Journal of Visualized Experiments	Inpatients, 299	Temperature: −20C, 4C or ambient temperature Time: Up to 14 days	Venipuncture, 100 μl whole blood on paper card	Not specified	Not specified	NR	NR	100%	NR	NR	NR
Marins, 2017 US	Journal of Virological Methods	48 patients with chronic HCV infection	Left to dry in ambient temperatures overnight, then in zip bags at ambient temperatures for 4 weeks	Venipuncture, 35 μl whole blood on Whatman card	COBAS® AmpliPrep/COBAS® Taqman® HCV Quantitative Test v2.0	COBAS® AmpliPrep/COBAS® Taqman® HCV Quantitative Test	Not detected at 4.75 log10 IU/mL.	NR	47/48 (98%)			
Marques Brazil 2016	Journal of Clinical Virology	99 (59 anti HCV/HCV RNA pos, 40 neg samples)	NR	Venipuncture, 3–5 drops on Whatman filter paper	In house	In house	58.5 copies/ml	100%	35/44 (65.9%)	Kappa: 0.648	Low variation during 3 days at ambient temperatures	NR
Mossner Denmark 2016	World Journal of Gastroenterology	404 Prospective patients from hepatitis clinic and blood donors	Temperature: Room temperature Time 1–5 days	Finger prick, 75 μ on Whatman filter paper	HCV RNA (Ultrio Elite dHBV). COBAS® AmpliPrep/COBAS® TaqMan® 48 system (Roche Diagnostics, Basel, Switzerland)	HCV RNA (Ultrio Elite dHBV). COBAS® AmpliPrep/COBAS® TaqMan® 48 system (Roche Diagnostics, Basel, Switzerland)	Limit of detection for HCV-RNA 100 IU/ml	25/26 96%	81/84 96.4%	NR	Variation of 24 h to up to 7 d found no difference in stability of samples	NR
Soulier, France 2016	The Journal of Infectious Diseases	511 patients recruited, with known serostatus for HCV	Temperature:-80 Time:	Venipuncture, 50 μl on Whatman filter paper	1) Cobas AmpliPrep automated extractor. The Cobas TaqMan 96 analyzer 2) m2000SP automated extractor	1) Cobas AmpliPrep automated extractor. The Cobas TaqMan 96 analyzer 2) m2000SP automated extractor	NR	1) 100% 196/196 2)100% 196/196	1) 97,1% 306/315 2) 98,1% 308/314	NR	25 dB samples stored at ambient temperatures (24 °C) for a mean duration (±SD) of 19 ± 1 months	

#### Assessment of study quality and risk of bias

Risk of bias assessments were limited by inadequate reporting. None of the studies reported blinding of laboratory personal to the results of reference/index tests. However, all studies used and reported a clear and consistent protocol for both reference and index test, so this was not judged as a major cause of bias. We downgraded the quality rating of four of the included studies because they used a case–control design, while the other five only sampled cases or did not adequately report on their sampling or the flow of participants. This resulted in an overall high risk of bias rating (Table 2). We did not perform a sensitivity analysis only for high-quality studies as too few studies would have been included, however high quality study results were generally consistent with overall results.

**Table 2:** Risk of bias in included studies for the systematic review on detection of HBV-DNA and update of the systematic review on detection of HCV-RNA from DBS samples compared to venous blood sampling (plasma/serum)

Author	Patient selection	Bias	Index test	Bias	Reference standard	Bias	Flow and timing	Bias
	Was a case control design avoided Consecutive or random sample of patients Inappropriate exclusions		Blinded to reference standard? Could the conduct or interpretation of the index test have introduced bias?		Are laboratory personelle blinded to index test? Could the reference standard have introduced bias?		Is there an appropriate interval between the index test and reference standard? All patients receive the same reference standard? All patients recruited into the study are included in the analysis?	
HBV-DNA								
Alidjinou	NR, but no case control design	UR	Not blinded, interpretation unbiased	LR	Not blinded, interpretation unbiased	LR	NR	UR
Alhusseini	Case control design, sampling NR	HR	Not blinded, interpretation unbiased	LR	Not blinded, interpretation unbiased	LR	NR	UR
Durgadevi	Case control design	HR	NR	UR	NR	UR	NR	UR
Gupta	Selection of only known HBV carriers,	HR	Not blinded, interpretation unbiased	LR	Not blinded, interpretation unbiased	LR	Partly reported	LR
Gruner	NR	UR	Not blinded, NR	UR	NR	UR	NR	UR
Jardi R	selection only of cases	HR	Not blinded, interpretation unbiased	LR	Not blinded, interpretation unbiased	LR	Partly reported	LR
Lira R	Selection of only cases	HR	Not blinded, interpretation unbiased	LR	Not blinded, interpretation unbiased	LR	NR	UR
Mohamed S	Case control design	HR	Not blinded, interpretation unbiased	LR	Not blinded, interpretation unbiased	LR	NR	UR
Mossner	Sampling from high-risk and low risk groups	HR	Not blinded, interpretation unbiased	LR	Not blinded, interpretation unbiased	LR	Sampling reported, same reference standard, all patients included in analysis	LR
Ross	No case control design, sampling NR	HR	Not blinded, interpretation unbiased	LR	Not blinded, interpretation unbiased	LR	Flow reported	LR
Stene- Johannsen	Sampling from high-risk	HR	Not blinded, interpretation unbiased	LR	Not blinded, interpretation unbiased	LR	Sampling reported, same reference standard, all patients included in analysis	LR
Vinikoor	No case control design, only cases	HR	Not blinded interpretation unbiased	LR	Not blinded, interpretation unbiased	LR	Flow reported	LR
HCV-RNA								
Dokubo	No case control, concurrent sampling from a prospective cohort	LR	NR	UR	NR	UR	Sampling reported, same reference standard, all patients recruited included in analysis	LR
Gruner	NR	UR	Not blinded, NR	UR	NR	UR	NR	UR
Marins	Only cases	HR	Not blinded, interpretation unbiased	LR	Not blinded, interpretation unbiased	LR	NR, same reference standard, NR	UR
Marques	NR	UR	Not blinded, interpretation unbiased	LR	Not blinded, interpretation unbiased	LR	NR, same reference standard, NR	UR
Mossner	Sampling from high-risk and low risk groups	HR	Not blinded, interpretation unbiased	LR	Not blinded, interpretation unbiased	LR	Sampling reported, same reference standard, all patients included in analysis	LR
Soulier	Sampling from high-risk and low risk-groups	HR	Not blinded, interpretation unbiased	LR	Not blinded, interpretation unbiased	LR	NR, same reference standard, NR	LR

*HR* High risk of bias, *LR* Low risk of bias, *UR* Unclear risk of bias, *NR* Not reported

#### Diagnostic performance

**Fig. 2:**
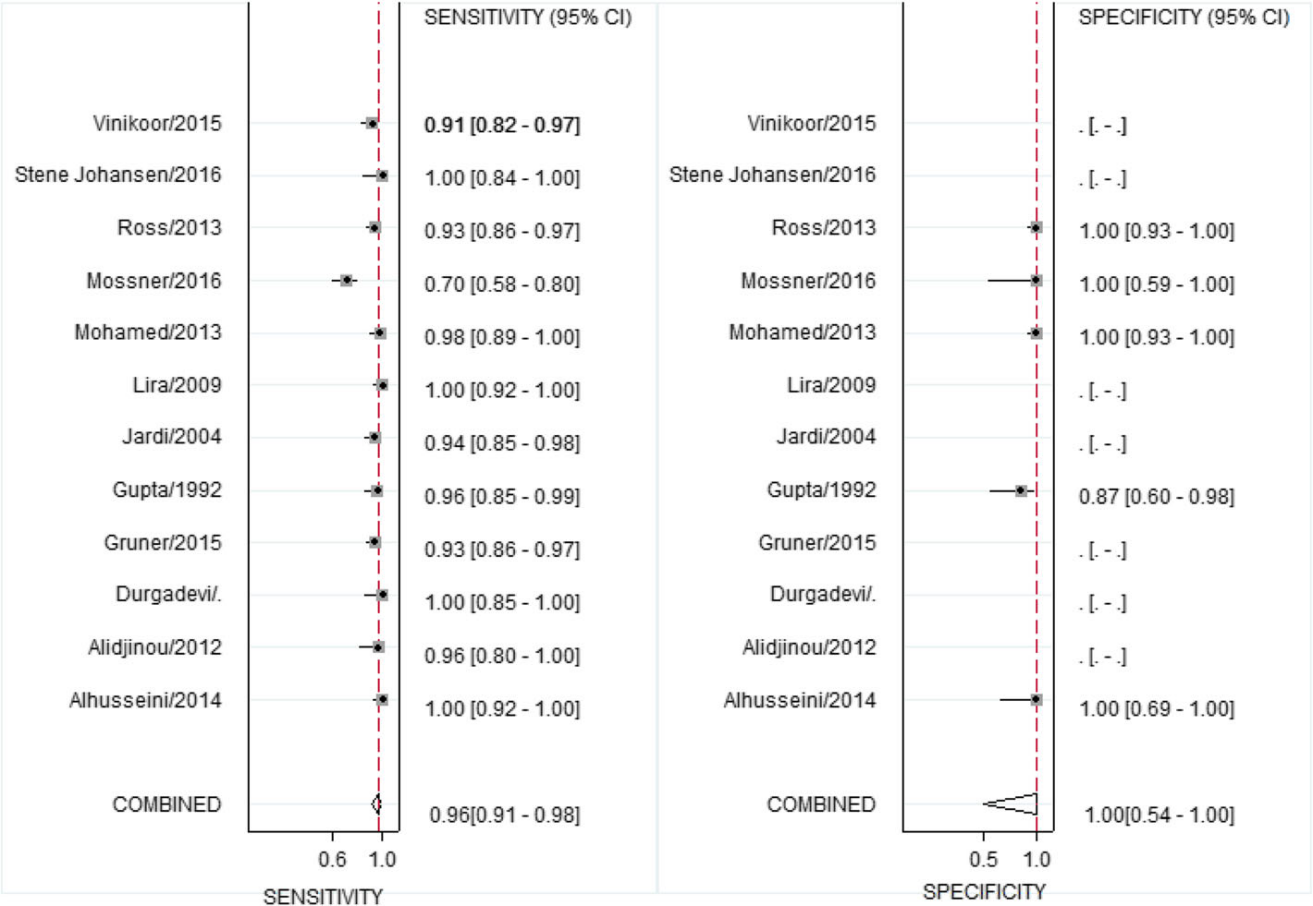
Forest plot of sensitivities and specificities of the use of DBS samples for detection of hepatitis B-DNA compared to serum samples

In the 5 studies that contributed to both sensitivity and specificity estimates, the pooled sensitivity of HBV-DNA using DBS ranged from 93 to 100% and specificity ranged from 70 to 100% (see Table 1). A pooled bivariate analysis estimated an overall sensitivity and specificity of 95% (95% CI%: 83–99%) and 99% (95% CI: 53–100%). A pooled univariate analysis yielded similar results for sensitivity (96%, 95% CI: 91–96, 12 studies;) and specificity (99%, 95% CI: 53–100, 5 studies) (Fig. 2). From the pooled sensitivity and specificity, the positive likelihood ratio was 379 (95% CI: 1–132,684) and the negative likelihood ratio was 0.05 (95% CI: 0.02–0.18). Visual assessment as a well as τ^2^ showed low heterogeneity (*p* = 0.1).

#### Association, correlation and agreement

Five studies reported regression coefficients showing a strong association between quantitative results of HBV-DNA in DBS and in serum [23–27] (regression coefficients between 0.61 to 0.96). One study reported a high Pearson correlation (0.93) [26] and another a high Spearman correlation coefficient of 0.84 [23].

#### Limit of detection (LoD) and cut-offs of HBV-DNA assays

The LoD of included assays (commercial and in-house) for serum samples ranged from 10 to 100 IU/ml (Table 1). The LoD using DBS in one study was 914 IU/ml for a commonly used commercial assay (COBAS Taqman), with a plasma-based limit of detection of 20 IU/ml [27]. Furthermore, two studies reported that quantitation of HBV-DNA in DBS below 3–4000 IU/ml was difficult [25, 33].

Only one study from Zambia reported on diagnostic accuracy of diluted samples at different cut-offs: − 14%(95% CI 8–24%) of those detected in plasma were missed using DBS at a cut-off below 200 IU/ml, 2% (95% CI: 0.5–7) at a cut-off below 2000 IU/ml (consistent with the lowest cut-off used for therapeutic indication) and 0.2% (95% CI: 0.03–1.7) at a cut-off below 20,000 IU/ml – the WHO recommended threshold for treatment [7]. Another recent study from Ethiopia showed decreased sensitivity of HBV DNA detection in samples with <2.7 log IU/ml [31] (Table 1).

#### Storage conditions, type of test and type of sample (DBS or DPS) used

For HBV-DNA detection, samples included in diagnostic accuracy estimates from three studies were stored at ambient temperatures for longer than 24 hours. One study stored 22 samples at ambient temperatures between 4 and 7 days and found no changes in detection of HBV-DNA with DBS after that time [29]. A second study stored 32 samples at ambient temperatures for an unknown time period, and calculated diagnostic accuracy from these [23]. A recently published study from Addis Ababa in Ethiopia demonstrated good diagnostic accuracy for detection and quantification of HBV-DNA and stability of samples in 21 samples under conditions that did not allow for refrigeration of samples over 12 weeks [31].

In addition, there were three studies that assessed for variation in results after differing duration of storage in ambient temperatures in subsets of DBS samples, that were not included in the overall diagnostic accuracy calculations. Two studies evaluated different storage conditions that ranged from 4 °C to 37 °C for up to 14 days and found no decrease in diagnostic accuracy [25, 26]. One study found no effect on stability of one sample after 5 months of storage at 37 °C [16].

We undertook a stratified univariate analysis for sensitivity and specificity according to whether an in-house PCR assay or a commercial assay was used. Pooled sensitivities were similar in both groups (95% and 98%) (data not shown). Only one study used plasma instead of whole blood for preparation of DBS samples, so a stratified analysis based on sample type was not possible. This single study showed similar sensitivity and specificity to the other studies and pooled estimates [24].

### Updated systematic review on HCV-RNA, 2013–2015

#### Included studies

**Fig. 3:**
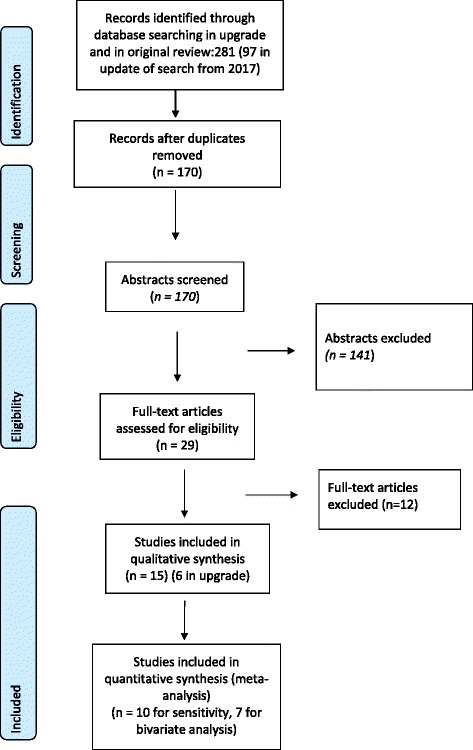
Prisma flow chart of studies for the systematic review of detection of hepatitis C RNA from DBS samples compared to venous blood sampling (plasma/serum)

We searched several databases (Medline, EMBASE, Global Health, Web of Science, Lilacs and the Cochrane database) with the same search terms as in the original systematic review [18]. After de-duplication, 97 articles were identified and underwent title and abstract review and six studies were included, that compared HCV-RNA detection using DBS to serum in adults in the US [34, 35], Europe [30, 32, 36] and in Brazil [37] (Fig. 3). In the previous systematic review, the 9 included studies [12, 38–45] of 73 identified titles, were from the UK, Italy, France, Lebanon, Brazil, Guinea-Bissau and Japan [18]. Three of the 15 studies were conducted in injecting drug users [12, 38].

#### Risk of bias

Overall risk of bias in the studies from the previous systematic review as well as the upgrade was rated as moderate [18, 20].

#### Diagnostic performance

**Fig. 4:**
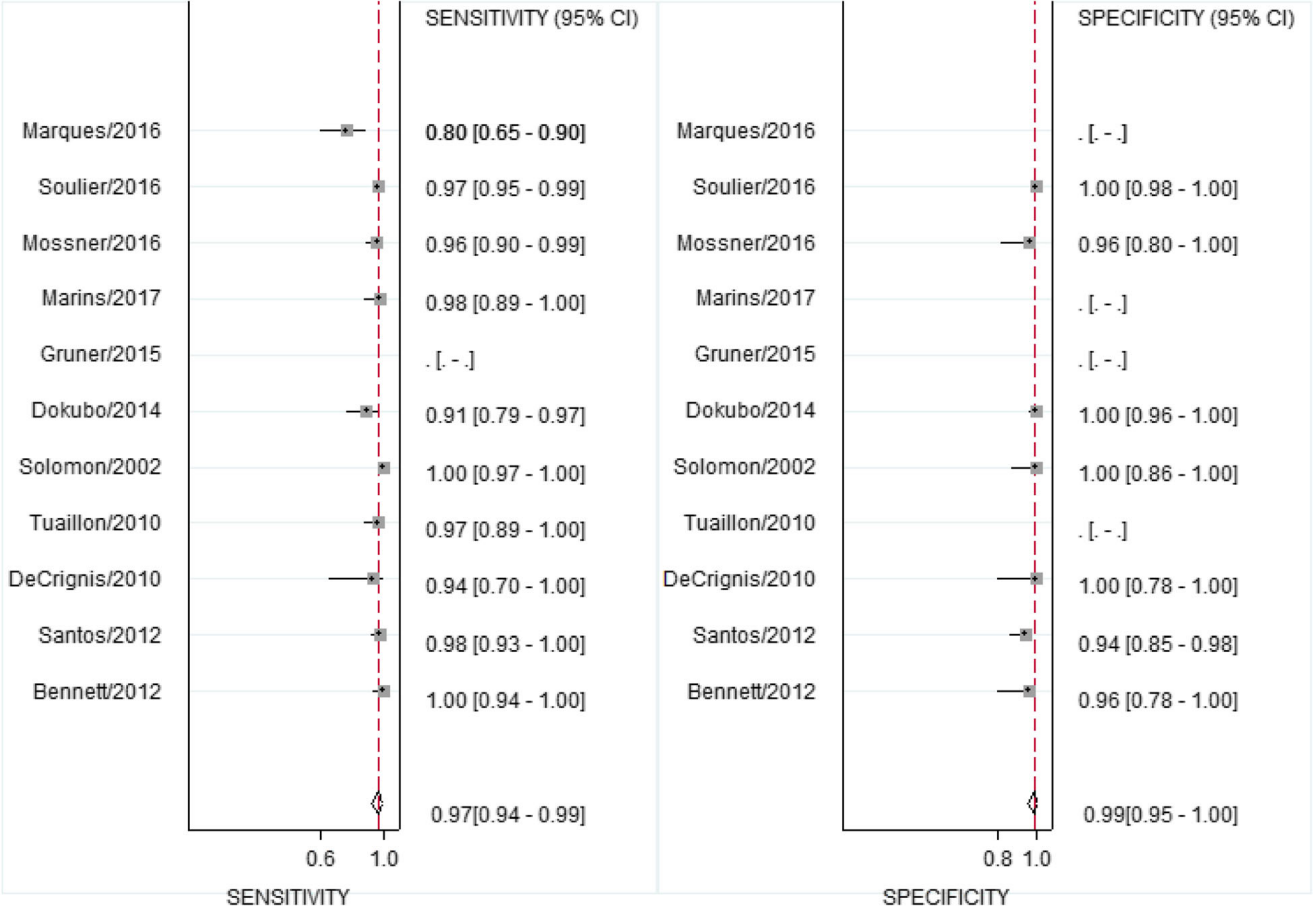
Forest plot of sensitivities and specificities of the use of DBS samples for detection of hepatitis C-RNA compared to serum samples

The additional studies included in the updated systematic review reported sensitivities between 80% to 100% and specificity between 94% and 100%. In the previously published systematic review five of nine studies provided data for a quantitative analysis. With inclusion of the additional studies in the upgraded review, pooled sensitivity was 98% (95% CI 95–99%) and specificity was 98%(95% CI:95–100%) (Fig. 4). Overall positive likelihood ratio was 63 (95% CI:22–181) and negative likelihood ratio was 0.02 (95% CI: 0.008–0.05).

### Assays and limit of detection (LoD) of HCV-RNA assays

The DBS samples in studies from the original systematic review were mainly prepared from capillary blood (8/9 studies) and a variety of commercially available and non-available methods were used to detect HCV RNA. A study from 2016 found a low limit of detection of 58.5 copies/ml for a newly developed quantitative RT PCR in DBS to amplify the 5’noncoding region of HCV [37]. Another study reported a limit of detection of DBS to be 150–250 IU/ml [39], while other studies reported higher limits of detection.

### Storage conditions and stability

Six HCV-RNA studies stored subsets of DBS specimens at ambient room temperature [30, 35, 39, 44, 45]. While these storage conditions did not affect accuracy, and RNA positivity could still be detected with DBS specimens, quantitative signals decreased over time in two studies. One study from France reported a 3-fold decrease in RNA levels after 6 days at room temperature in a subset of an unknown number of samples. A second study from Japan found a reduction in viral load level, but no reduction in detection of positivity after 4 weeks of ambient temperatures [44]. Two other studies did not find decrease in HCV-RNA quantification from DBS samples after prolonged (>11 months) periods at room temperature [39, 45].

## Discussion

This is the first systematic review to report on the diagnostic performance of DBS for HBV DNA, together with an updated systematic review for HCV RNA measurement. The reviews were based on 12 and 15 studies respectively and overall show a high level of diagnostic accuracy with sensitivity and specificity for HBV-DNA of 96% and 99% and for HCV-RNA of 98% and 98% using DBS. However, these systematic reviews were based on a relatively small number of studies compared to the pooled analysis of DBS for HBsAg and HCV antibody serology [19] and were rated as low quality evidence with significant risk of bias. In addition, for HBV-DNA detection, there was a high degree of imprecision for the pooled estimate of specificity 99% (95% CI: 54–100%). A narrative review in a subset of studies showed a good correlation and strong association between quantitative values for HBV-DNA from DBS and in serum samples.

Most of the individual studies suggest that the sensitivity of HBV-DNA detection using DBS above 2000 IU/mL is good, and the LoDs in DBS specimens is 900–4000 IU/mL. This means that tests using DBS samples will be able to identify the majority of persons with chronic hepatitis B infection in need of antiviral treatment.

While all guidelines agree on the need to treat above a cut-off of 20,000 IU/mL, there is some divergence in treatment recommendations between 2000 and 20,000 IU/mL [5, 7–9]. The American Association for the Study of Liver Disease recommends initiating antiviral treatment in non-pregnant adults with chronic HBV but without cirrhosis based on either an HBV DNA above 2000 IU/ml or above 20,000 IU/ml in those patients with either above twofold ALT increase or with histological disease [5]. The European Association of the Study of Liver and the latest WHO guidelines concur on a HBV cut-off of >2000 IU/ml (without cirrhosis) [7, 9]. As DBS has not yet been evaluated for monitoring of treatment response, and the cut-off for detection is not precisely known, further studies on DBS specimens are needed to evaluate their validity in monitoring HBV-DNA response to treatment and viral suppression with long-term therapy.

In the evaluation of the diagnostic accuracy of HCV RNA measurement using DBS, six further recent studies were added to the nine already identified in a previous systematic review [18] and a pooled quantitative analysis of diagnostic accuracy was also undertaken which was not part of the initial review (including 10 studies). A quantitative viral load is no longer essential to confirm presence of HCV viraemic infection and therefore need for treatment in those with positive HCV antibody serology, or to monitor response to DAA treatment. Determination of the presence of virus at diagnosis can be based on a qualitative assay alone, and indeed the 2017 WHO testing guidelines recommended either a qualitative or quantitative NAT assay for diagnosis of viraemic HCV infection. This is because the new generation of quantitative and qualitative assays have the same LoD, which is around 15 IU/ml, and data show that 95% of those with chronic infection have a viral load >10,000 IU/mL, and therefore most NAT assays (quantitative or qualitative) will capture the majority of viraemic infections [20]. Those studies reporting LoD in the systematic review for detection of HCV RNA with DBS samples showed LoDs between 50 and 250 IU/ml, which is higher but would still be sufficient for detection of the majority of infections.

This review did not assess the use of DBS specimens to assess response to HCV direct acting antiviral (DAA) treatment and proof of cure because of the lack of studies that have specifically addressed this question. However, as the recommended test of cure is attainment of sustained virologial response after 12 weeks (or 24 weeks), as for diagnosis, the need for quantification is diminished [18, 46]. Preliminary evidence suggests that patients failing DAAs have high viral loads at 12 or 24 weeks post treatment completion, implying that testing with DBS may be feasible. Although the cut-offs at which HCV-RNA can be detected using DBS specimens are not well characterized, the evidence suggests that qualitative detection of HCV-RNA using DBS specimens is possible and accurate.

In assessing the generalizability of the pooled results for use of DBS for HBV-DNA measurement, it is noteworthy that only two of the included studies stored dried plasma and blood samples at ambient temperatures [23, 29]. In the other 7 included studies, the samples were refrigerated or frozen. This may limit applicability to field conditions likely to be encountered in low resource settings, where it may not be possible to refrigerate or freeze samples locally or during transport. Several other studies assessed subsets of samples exposed to varying storage conditions and found no effect on the qualitative result of these assays [25–27]. Overall we did not undertake a formal pooled analysis of the effect of refrigerated storage (due to low number of studies) but based on the narrative review, the studies that reported using refrigerated samples had a similar diagnostic accuracy to the two studies that reported storing samples at ambient temperatures.

We also examined the impact of type of assay (commercial vs in-house) and use of a case–control design and found no differences in diagnostic accuracy (data not shown). Study quality overall was rated as moderate, mainly due to underreporting of important sources of bias.

Of the fifteen included studies in the original review and this upgrade, six HCV-RNA studies stored DBS specimens at ambient temperature [18, 30, 35, 44, 45]. In the previous systematic review [18], three studies had exposed samples to a variety of temperatures and storage conditions, and RNA positivity could still be detected with DBS, but with a decrease in the quantitative signals over time [42, 44, 45].

The key limitations of these systematic reviews were first, the overall small number of studies; second, that non-English or unpublished studies were excluded, and third, the overall low rating of study quality. In addition, we were not able to ascertain the cut-offs for detection of HBV-DNA and HCV-RNA detection DBS samples and whether results remained valid with storage at ambient temperatures for prolonged time periods.

## Conclusions

The principal benefits of DBS specimens is their potential to increase access to testing [10], largely because of the simplicity and relative ease of specimen collection, preparation and transport as it avoids the need for plasma separation within a specified time period, and cold chain storage. Based also on the evidence from these two systematic reviews, the WHO 2017 testing guidelines conditionally recommended use of DBS specimens as an option for both HBV and HCV NAT, especially in settings where there are no facilities or expertise to take venous blood specimens, or for persons with poor venous access [20].

### Future research

There is now a need for larger and more rigorously-performed DBS diagnostic accuracy studies. These include studies that avoid case control designs, test HBV-DNA and HCV-RNA DBS under a range of real-life storage and transport conditions (e.g. room temperature for different duration, duration of subsequent storage at −20° versus −80°, use of individual plastic bags with dessicant) and report diagnostic accuracy for different time, volume of whole blood used for testing temperature and clinically relevant LoD cut-offs. Manafacturers also need to validate the use of their assays with DBS specimens and to standardize technical guidance regarding their use. There is also a need for evaluation of the impact of HIV positivity and immunosuppression on HBV DNA and HCV RNA diagnostic performance using DBS. The use of DBS should also be validated for monitoring of treatment response and HCV test of cure (SVR at 12 or 24 weeks) post DAA therapy, including threshold for detection. This includes validation of the rate of degradation of HCV RNA and detectability when stored at ambient temperatures and high humidity for different time periods. Finally, there remains limited programmatic experience in the use of DBS for hepatitis testing across a wide range of testing settings in low and middle-income countries and its impact on uptake of testing, identification of cases of HBV and HCV and linkage to treatment.

## References

[1] Gower E, Estes C, Blach S, Razavi-Shearer K, Razavi H (2014). Global epidemiology and genotype distribution of the hepatitis C virus infection. J Hepatol.

[2] Stanaway JD, Flaxman AD, Naghavi M, Fitzmaurice C, Vos T, Abubakar I (2016). The global burden of viral hepatitis from 1990 to 2013: findings from the global burden of disease study 2013. Lancet.

[3] World Health Organization. Global Hepatitis Report. Geneva: World Health Organization; 2017.

[4] Omata M, Kanda T, Wei L, M-L Y, Chuang W-L, Ibrahim A (2016). APASL consensus statements and recommendations for hepatitis C prevention, epidemiology, and laboratory testing. Hepatol Int.

[5] American Association for the Study of Liver Disease. Recommendations for Testing, Managing, and Treating Hepatitis C. AASLD IDSA; 2015. https://www.hcvguidelines.org/.

[6] Sarin SK, Kumar M, Lau GK, Abbas Z, Chan HLY, Chen CJ (2016). Asian-Pacific clinical practice guidelines on the management of hepatitis B: a 2015 update. Hepatol Int.

[7] World Health Organization. Guidelines for the prevention, care and treatment of persons with chronic hepatitis B infection. Geneva: World Health Organization; 2015. http://www.who.int/hiv/pub/hepatitis/hepatitis-b-guidelines/en/. 26225396

[8] NICE. Diagnosis and management of chronic hepatitis B in children, young people and adults. 2013. https://www.nice.org.uk/guidance/cg165. 25473721

[9] European Association For The Study Of The Liver (2017). EASL clinical practice guidelines: clinical practice guidelines on the management of hepatitis B virus infection. J Hepatol.

[10] Snijdewind IJM, van Kampen JJA, Fraaij PLA, van der Ende ME, Osterhaus ADME, Gruters RA (2012). Current and future applications of dried blood spots in viral disease management. Antivir Res.

[11] Komas NP, Vickos U, Hubschen JM, Bere A, Manirakiza A, Muller CP (2013). Cross-sectional study of hepatitis B virus infection in rural communities, Central African Republic. BMC Infect Dis.

[12] Mahfoud Z, Kassak K, Kreidieh K, Shamra S, Ramia S (2010). Prevalence of antibodies to human immunodeficiency virus (HIV), hepatitis B and hepatitis C and risk factors in prisoners in Lebanon. J Infect Dev Ctries.

[13] Costa ZB, Machado GC, Avelino MM, Gomes Filho C, Macedo Filho JV, Minuzzi AL (2009). Prevalence and risk factors for hepatitis C and HIV-1 infections among pregnant women in Central Brazil. BMC Infect Dis.

[14] Dube Q, Dow A, Chirambo C, Lebov J, Tenthani L, Moore M (2012). Implementing early infant diagnosis of HIV infection at the primary care level: experiences and challenges in Malawi. Bull World Health Organ.

[15] Smit PW, Sollis KA, Fiscus S, Ford N, Vitoria M, Essajee S (2014). Systematic review of the use of dried blood spots for monitoring HIV viral load and for early infant diagnosis. PLoS One.

[16] Gupta BP, Jayasuryan N, Jameel S (1992). Direct detection of hepatitis B virus from dried blood spots by polymerase chain reaction amplification. J Clin Microbiol.

[17] Smit PW, Elliott I, Peeling RW, Mabey D, Newton PN (2014). Review article: an overview of the clinical use of filter paper in the diagnosis of tropical diseases. Am J Trop Med Hyg.

[18] Greenman J, Roberts T, Cohn J, Messac L (2015). Dried blood spot in the genotyping, quantification and storage of HCV RNA: a systematic literature review. J Viral Hepat.

[19] Lange B, Cohn J, Roberts T, Camp J, Chauffour J, Gummadi N, et al. Diagnostic accuracy of serological diagnosis of hepatitis C and B using dried blood spot samples (DBS): two systematic reviews and meta-analyses. BMC Infect Dis. 2017;17(1). Suppl doi:10.1186/s12879-017-2777-y. 10.1186/s12879-017-2777-y PMC5688450 29143672

[20] World Health Organization. Guidelines on Hepatitis B and C Testing. Geneva: World Health Organization; 2017. http://www.who.int/hepatitis/publications/guidelines-hepatitis-c-b-testing/en/.

[21] Whiting PF, Rutjes AW, Westwood ME, Mallett S, Deeks JJ, Reitsma JB (2011). QUADAS-2: a revised tool for the quality assessment of diagnostic accuracy studies. Ann Intern Med.

[22] Naaktgeboren CA, Ochodo EA, Van Enst WA, de Groot JAH, Hooft L, Leeflang MMG (2016). Assessing variability in results in systematic reviews of diagnostic studies. BMC Med Res Methodol.

[23] Alidjinou EK, Moukassa D, Sane F, Nyenyeli ST, Akoko EC, Mountou MV (2014). Detection of hepatitis B virus infection markers in dried plasma spots among patients in Congo-Brazzaville. Diagn Microbiol Infect Dis.

[24] Alhusseini NF, Abadeer MZ, El-Taher SM, Hepatitis B (2012). Virus DNA can be amplified directly from dried blood spot on filter paper. Am J Biochem Biotechnol.

[25] Jardi R, Rodriguez-Frias F, Buti M, Schaper M, Valdes A, Martinez M (2004). Usefulness of dried blood samples for quantification and molecular characterization of HBV-DNA. Hepatology.

[26] Lira R, Maldonado-Rodriguez A, Rojas-Montes O, Ruiz-Tachiquin M, Torres-Ibarra R, Cano-Dominguez C (2009). Use of dried blood samples for monitoring hepatitis B virus infection. Virol J.

[27] Mohamed S, Raimondo A, Penaranda G, Camus C, Ouzan D, Ravet S (2013). Dried blood spot sampling for hepatitis B virus serology and molecular testing. PLoS One.

[28] Vinikoor MJ, Zurcher S, Musukuma K, Kachuwaire O, Rauch A, Chi BH (2015). Hepatitis B viral load in dried blood spots: a validation study in Zambia. J Clin Virol.

[29] Durgadevi S, Dhodapkar R, Parija SC. Serological and molecular diagnosis of hepatitis B virus. BMC Infect Dis Conference: 1st International Science Symposium on HIV and Infectious Diseases, HIV SCIENCE. 2012;20120120(20120122).

[30] Mossner BK, Staugaard B, Jensen J, Lillevang ST, Christensen PB, Holm DK (2016). Dried blood spots, valid screening for viral hepatitis and human immunodeficiency virus in real-life. World J Gastroenterol.

[31] Stene-Johansen K, Yaqoob N, Overbo J, Aberra H, Desalegn H, Berhe N (2016). Dry blood spots a reliable method for measurement of hepatitis B viral load in resource-limited settings. PLoS One.

[32] Gruner N, Stambouli O, Ross RS. Dried blood spots--preparing and processing for use in immunoassays and in molecular techniques. J Visual Exp. 2015;97 10.3791/52619 PMC4397000 25867233

[33] Ross RS, Stambouli O, Gruner N, Marcus U, Cai W, Zhang W (2013). Detection of infections with hepatitis B virus, hepatitis C virus, and human immunodeficiency virus by analyses of dried blood spots--performance characteristics of the ARCHITECT system and two commercial assays for nucleic acid amplification. Virol J.

[34] Dokubo EK, Evans J, Winkelman V, Cyrus S, Tobler LH, Asher A (2014). Comparison of hepatitis C virus RNA and antibody detection in dried blood spots and plasma specimens. J Clin Virol.

[35] Marins EG, Bodinaidu K, Lin M, Deforest A (2017). Evaluation of the COBAS(R) AmpliPrep/COBAS(R) TaqMan(R) HCV test v2.0 for HCV viral load monitoring using dried blood spot specimens. J Virol Methods.

[36] Soulier A, Poiteau L, Rosa I, Hezode C, Roudot-Thoraval F, Pawlotsky JM (2016). Dried blood spots: a tool to ensure broad access to hepatitis C screening, diagnosis, and treatment monitoring. J Infect Dis.

[37] Marques BL, do Espirito-Santo MP, Marques VA, Miguel JC, da Silva EF, Villela-Nogueira CA (2016). Evaluation of dried blood spot samples for hepatitis C virus detection and quantification. J Clin Virol.

[38] Hope VD, Hickman M, Ngui SL, Jones S, Telfer M, Bizzarri M (2011). Measuring the incidence, prevalence and genetic relatedness of hepatitis C infections among a community recruited sample of injecting drug users, using dried blood spots. J Viral Hepat.

[39] Bennett S, Gunson RN, McAllister GE, Hutchinson SJ, Goldberg DJ, Cameron SO (2012). Detection of hepatitis C virus RNA in dried blood spots. J Clin Virol.

[40] Santos C, Reis A, Dos Santos CV, Damas C, Silva MH, Viana MV (2012). The use of real-time PCR to detect hepatitis C virus RNA in dried blood spots from Brazilian patients infected chronically. J Virol Methods.

[41] De Crignis E, Re MC, Cimatti L, Zecchi L, Gibellini D (2010). HIV-1 and HCV detection in dried blood spots by SYBR green multiplex real-time RT-PCR. J Virol Methods.

[42] Tuaillon E, Mondain AM, Meroueh F, Ottomani L, Picot MC, Nagot N (2010). Dried blood spot for hepatitis C virus serology and molecular testing. Hepatology.

[43] Plamondon M, Labbe AC, Frost E, Deslandes S, Alves AC, Bastien N (2007). Hepatitis C virus infection in Guinea-Bissau: a sexually transmitted genotype 2 with parenteral amplification?. PLoS One.

[44] Abe K, Konomi N, Hepatitis C (1998). Virus RNA in dried serum spotted onto filter paper is stable at room temperature. J Clin Microbiol.

[45] Solmone M, Girardi E, Costa F, Ippolito G, Capobianchi M (2002). Simple and reliable method for HCV-RNA detection/genotyping in dried blood spots. J Hepatol.

[46] Cohn J, Roberts T, Amorosa V, Lemoine M, Hill A (2015). Simplified diagnostic monitoring for hepatitis C, in the new era of direct-acting antiviral treatment. Curr Opin HIV AIDS.

